# Application of water-soluble conjugated polymers in intelligent measurement and control of food microbial fermentation process

**DOI:** 10.3389/fchem.2023.1269907

**Published:** 2023-09-15

**Authors:** Chang Liu, Yujiao Tang

**Affiliations:** ^1^ School of Life Sciences, Changchun Sci-Tech University, Jilin, China; ^2^ Jilin Sino-ROK Institute of Animal Science, Jilin, China

**Keywords:** water-soluble conjugated polymers, food science, microbial fermentation, biological sensors, measurement and control systems

## Abstract

In order to reduce the difficulty of measurement and control (MAC) during food fermentation, this paper applies water-soluble conjugated polymers to sensors, conducts data modeling and prediction, and integrates the sensors into intelligent MAC systems. This article uses fermentation rate, product yield, and energy consumption efficiency as evaluation indicators to analyze the effectiveness of smart MAC. By comparing and analyzing the MAC method based on water-soluble conjugated polymers with the traditional MAC method, this article found that the MAC method based on water-soluble conjugated polymers can improve product yield, fermentation efficiency and energy utilization compared with traditional MAC methods. The MAC accuracy, timeliness, stability, speed and security of the MAC system based on water-soluble conjugated polymers are higher than those of traditional MAC systems. Among them, the average test stability of the traditional MAC system is 19.93% lower than that of the smart MAC system based on water-soluble conjugated polymers. Research shows that the intelligent MAC method based on water-soluble conjugated polymers can effectively improve the fermentation environment of food microorganisms and improve product quality, and is worthy of further promotion.

## 1 Introduction

Microbial fermentation is the foundation of bioengineering, modern biotechnology, and industrialization (AO et al., 2020; Gargalo et al., 2020). With the continuous development of fermentation technology, the demand for intelligent MAC technology is also increasing. However, the mechanism of biological fermentation process is complex and highly nonlinear, and changes over time (Villarreal-Soto et al., 2018). Some key parameters (such as substrate concentration, bacterial concentration, and product yield) are difficult to detect online, and it is also difficult to implement advanced optimization algorithms and management strategies. At present, most fermentation systems based on different MAC units use traditional controllers, which do not have intelligent components to detect key parameters. With the development and progress of the times, the application of conjugated polymers has provided certain technical support for the MAC of fermentation processes.

The MAC of food microbial fermentation process is often carried out through the construction of a system. Zhang Yaju designed a MAC system for the fermentation process of penicillium pentosum based on CO2 gas concentration feedback control feeding. This system achieved online detection of CO2 gas concentration during the fermentation process through electrochemical gas sensors. It utilized OPC (Open Platform Communications) technology and Lab VIEW programming to obtain real-time data on CO2 gas concentration during the fermentation process and feedback control feeding during the fermentation process. Combined with PLC (Programmable Logic Controller), it optimized and regulated the fermentation of *streptococcus* pentosus. After testing, the system has achieved real-time monitoring and online feedback control of CO2 gas concentration during the fermentation process of *streptococcus* pentosus, verifying the feasibility and reliability of the system (Zhang et al., 2019). Bai Yunsong has established a complete set of environmental parameter model of Daqu starter room, and analyzed the environmental temperature distribution and change rule of Daqu starter room during the fermentation process (Bai et al., 2019). Although the method of constructing MAC systems during the fermentation process has been widely applied, few people have utilized water-soluble conjugated polymers for fermentation MAC.

Fat soluble conjugated polymers are usually soluble in organic solvents, while water-soluble conjugated copolymers are made of lipophilicity polymers. They can be dissolved in water by functionalization of side chains of lipophilicity conjugated polymers and modification of hydrophilic groups. Hydrophilic groups include pyridine cation, sulfonic acid, carboxylate, phosphate, and other groups. Water-soluble conjugated polymers combine the solubility of polyelectrolyte in water and the photoelectric properties of conventional conjugated polymers, and are widely used in biological detection fields, such as detection of organic small molecules and metal cations, DNA (deoxyribonucleic acid) and proteins. They are a type of linear polymer compounds with large π-π* conjugated electronic structures on the main chain, which can modify hydrophilic groups on the side chains (Zhou et al., 2019). It not only has high quantum yield, stable optical properties, significant fluorescence enhancement, and strong quenching effect, but also has good water solubility and strong electrostatic effect. Based on these advantages, water-soluble conjugated polymers are widely used as biochemical sensors, achieving high sensitivity of metal ions, biomolecules, and organic small molecules. Conjugated polymers are polymers composed of alternating C-C single bonds, C=C double bonds, and C≡C triple bonds on the main chain. The isolated electronic bonding system allows electrons or energy to move freely around the conjugated framework, thus possessing the function of “molecular wires”, which can produce polymers from traditional insulators, semiconductors, and even conductors.

The structure of water-soluble conjugated polymers determines their unique photochemical and photophysical properties (Lu et al., 2018; Dai and Liu, 2020). The soluble conjugated polymer structure is divided into two parts: 1) a hydrophobic framework with an out of band electronic structure. It determines the light absorption or emissivity, signal speed and quantum fluorescence properties of conjugated polymers. According to the main chain, conjugated polymers can be divided into polythiophene, polyfluorene, poly (p-phenylene vinylidene), poly(arylene ethynylene), polypyrrole, polyphenylene, poly (diacetylene) and polyfluorene benzene. 2) The side chains connected to the hydrophobic backbone: it can significantly increase the solubility of conjugated polymers in water, thereby regulating their interactions with biological macromolecules, cells, or bacteria. In addition, water-soluble conjugated polymers with special functions can bind through the functional end of side chains, such as drug molecules, photosensitive substances, specific controllers, etc.

Based on the various advantages of water-soluble conjugated polymers, they have excellent application prospects in intelligent MAC of food microbial fermentation processes. Based on this, this article applies water-soluble conjugated polymers to the design and implementation of sensors to detect substances such as glucose, protein, and sugars during the fermentation process. Based on the collected signals, the fermentation process is warned and people are reminded to adjust fermentation parameters in a timely manner, thus increasing the fermentation rate. Comparing and analyzing the MAC methods based on water-soluble conjugated polymers in this article with traditional methods, it is found that the system performance of the MAC method based on water-soluble conjugated polymers in this article is good, and it has excellent fermentation advantages in the process of food microbial fermentation.

## 2 Application and implementation of water-soluble conjugated polymers

### 2.1 Sensor design and preparation

#### 2.1.1 Selection of water-soluble conjugated polymer materials

Water-soluble conjugated polymer materials were selected to utilize a novel bioprobe technology based on water-soluble fluorescent conjugated polymers: quencher-tether-ligand (QTL), which is a fast, simple, sensitive, and selective homogeneous analysis method (Song et al., 2020). QTL consists of three parts: quenching agent, connecting arm, and receiver. The receiver can selectively bind to the substance to be tested. Both ends of the connecting arm are connected to the receiver and quenching agent. The quenching agent has weak coordination with the fluorescent polymer, resulting in fluorescence quenching. When certain viruses, chemical molecules, or biological molecules exist, they interact with compounds in a strong and specific manner and restore fluorescence. Sensitive detection of chemical substances or biological molecules can be achieved by observing changes in fluorescence intensity.

Biosensors can convert biomolecular detection into measurable signals, while water-soluble polymers can increase the signal of interaction with biomolecules, greatly improving detection sensitivity (Sireesha et al., 2018; Cao et al., 2019; Reid et al., 2020; Lv et al., 2022). They can detect interactions between probe molecules and matrix molecules through fluorescence resonance energy transfer, electron transfer, or induced changes in matrix conformation (Abdullah, , 2022; Cao et al., 2022a; Cao et al., 2022b). Fluorescence resonance energy transfer is when two fluorescent chromosomes are close enough, and when the donor molecule absorbs photons of a specific frequency and excites them to a higher electronic state, before the electrons return to the basic state, the energy is transferred to adjacent receptor molecules through dipole interaction. According to the energy transfer formula, the resonance energy transfer efficiency fluorescence depends on the overlap of donor radiation spectrum and receptor absorption spectrum, the relative direction between donor dipole and excited state receptor, and the distance between donor and receptor molecules. If the donor and receptor molecules meet the above conditions, the donor molecule can be activated and resonance energy can be transferred from the donor to the receptor (Kanvita, 2021; Radhakrishnan, 2022). Therefore, when selecting conjugated polymers as signal converters in this article, it is necessary to consider whether the radiation spectra of the polymers and the absorption spectra of the receptor molecules basically overlap. The relative orientation and distance between conjugated polymer molecules and receptor molecules can achieve optimal energy transfer efficiency fluorescence for resonance.

Based on the above conditions, this article designs a highly sensitive hydrogen peroxide probe using cationic polyfluorene fluorescence quenching, and a glucose sensor using hydrogen peroxide as the signal carrier. On this basis, a water-soluble conjugated polymer containing borate ester to protect fluorescein, hydrogen peroxide, and glucose is designed and synthesized for the detection of hydrogen peroxide and glucose in fermentation broth using fluorescence ratio. Because the most important application link in current fermentation engineering is the use of yeast fermentation to produce wine, detecting glucose can greatly improve the fermentation rate of wine.

#### 2.1.2 Establishment of evaluation indicators

This article selects fermentation rate, product yield, and energy efficiency as evaluation indicators for intelligent MAC of food microbial fermentation processes. Differentiating the evaluation methods of various evaluation indicators: the testing of fermentation rate evaluates the impact of intelligent MAC systems on fermentation rate by monitoring the rate of change of key indicators. The testing of product yield compares the quality differences between intelligent MAC systems and traditional methods, and evaluates their impact on product yield. The testing of energy efficiency considers the energy consumption of intelligent MAC systems during implementation, and evaluates their impact on energy utilization efficiency.

#### 2.1.3 Application of sensors in intelligent MAC of food microbial fermentation process

In the process of microbial fermentation of food, glucose is measured, and ethanol concentration and cell concentration are also measured, in order to technically observe the changes of ethanol and microbial cell concentration in the fermentation broth, so as to control the process of fermentation in time (Morales and Halpern, 2018; Ole et al., 2021). Therefore, in the application of food microbial fermentation process, this article has designed the detection of glucose content, ethanol concentration, and microbial cell concentration.

Glucose content detection: during fermentation, the growth rate of microorganisms can be determined based on sugar consumption, observing whether they are contaminated, estimating the conversion rate together with the production of products at any time, determining the feeding effect, and timely judging the end time of fermentation. Abnormal phenomena during the fermentation process or equipment can be timely predicted through glucose analysis.

**FIGURE 1:**
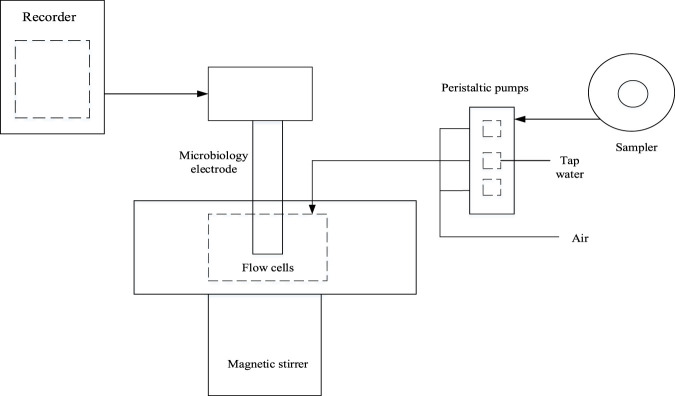
Traditional ethanol sensor.

Ethanol concentration detection: directly monitoring the ethanol content in fermentation broth is a commonly used method for monitoring the microbial fermentation process of food, which is very important for the fermentation industry. When sugar is used as a carbon source for yeast culture, ethanol is a byproduct, and the consumption of sugar is proportional to the content of yeast cells. When methanol is used as a carbon source for microbial growth, its concentration must be controlled to avoid the inhibitory effect of methanol on microorganisms. The respiratory activity of microorganisms reflects their utilization of digestible organic compounds, which can be measured through anodes. Thus, a probe comprising a microorganism capable of utilizing ethanol or methanol and an oxygen electrode can be used to determine the amount of ethanol or methanol. This probe can be used to continuously measure the amount of ethanol or methanol in fermentation broth. Methanol and ethanol sensors are usually prepared using methanol bacteria and *saccharomyces cerevisiae*. When the sample solution containing ethanol enters the measurement system, ethanol passes through the permeation membrane and is utilized by microorganisms. At this point, the dissolved oxygen around the membrane decreases, causing the current to decrease significantly until it reaches a stable state. The schematic diagram of the traditional ethanol sensor can be seen in Figure 1:

Microbial cell concentration measurement: there are two main types of microbial sensors. One is a sensor for measuring microbial respiration, which uses an electrochemical device to measure changes in the respiratory activity of fixed microorganisms. Another type is a microbial electroactive substance measurement sensor, which uses oxygen electrodes, fuel cell electrodes, carbon dioxide gas electrodes, or ion electrodes in electrode devices to measure the electroactive substances produced by microorganisms on simple reactant electrodes such as hydrogen, formic acid, hydrogen ions, and coenzymes. Measuring the concentration of microbial cells in a fermentation tank is crucial for controlling the fermentation process. The commonly used method is the optical density method. However, this method is time-consuming and cannot be continuously measured. This article requires a simple and continuous cell counting method to control the fermentation process. Because some bacteria can directly oxidize on the anode surface to generate current, this electrochemical principle can be used to determine the number of cells. The microbial cell concentration sensing system used in this article consists of a measurement section and a control section. Each part contains a working electrode, a counting electrode and a saturated electrode, with saturated potassium chloride solution as the electrolyte. Three electrodes are connected to each other through one electromotive force meter, and the current is measured using a millivoltmeter and recorded using a recorder. By measuring the electromotive force of two parts and their potential impact on the generation of different currents, the concentration of microbial cells can be determined.

### 2.2 Signal acquisition and processing

The data acquisition of the intelligent MAC system for food microbial fermentation process based on water-soluble conjugated polymers designed in this paper is an integrated technology based on analog signal processing, digitization, digital signal processing and computer technology. In a monitoring system, the data collection system consists of sensors, fieldbus, and front-end processors. In the fermentation control system, parameters are measured by reading signals of fermentation tank temperature, pH, dissolved oxygen, and ethanol concentration. During the fermentation process, the measurement results are transmitted to the MAC system.

By analyzing the collected data, the trend of each parameter can be determined. After processing the relevant data, the microcomputer sends the control signal, which is received by the transmitter (peristaltic pump and air pump) after D/A (Digital to Analog Converter) conversion. The actuating device is then subjected to appropriate liquid or air replenishment operations to obtain optimized control of the fermentation reaction.

The system enhances the concentrated signal by using the high pure suppression ratio of the instrument amplifier, effectively eliminating pure interference. The use of low-pass band-resistant hybrid filters makes it possible to filter noise outside the signal band. The processed A/D (Analog to Digital Converter) data is processed by a microcontroller and transmitted to the main computer through a USB (universal Serial Bus) interface. The main computer displays and stores measurement data in real-time through a human-machine interaction interface based on LabVIEW (Laboratory Virtual Instrument Engineering Workbench).

#### 2.2.1 Amplification circuit design

The excitation voltage of the load converter for the biosensor selected in this article is 6V. The MP2451 is selected for voltage reduction and circuit stabilization to obtain a stable excitation voltage at 24V external voltage. MP2451 is a high-frequency voltage regulator with fast ring start and efficient output current control mode. The stable excitation voltage effectively reduces the zero thermal drift of the voltmeter. After the voltage is reduced, the current output is about 40mA to ensure that the voltmeter operates at the rated current, reduce the thermal effect, and achieve the best temperature compensation.

The biosensor selected in this article adopts a fully straight circular bridge arm, which has high sensitivity and good linearity, and is easy to achieve temperature compensation. Installing a gain circuit below the sensor prevents amplification of certain parasitic signals during transmission to the sampling interface A/D. When the stable voltage source is activated, the bridge outputs a differential signal of mV level variation under the excitation of a 6V stabilized voltage source, with R7 adjustable to zero. The resistor is set to R12 to obtain the desired amplification gain and full scale output. This amplification circuit has strong common mode suppression ability, and the amplified signal voltage range varies from 0V to 3.3V.

#### 2.2.2 Main control board circuit design

The enhanced signal is transmitted to the control panel through aluminum foil and grid signal lines, but it may also affect electromagnetic interference in other parts of the system. Therefore, it is necessary to filter the signal within the range of 0.05Hz to 1kHz and ensure that the signal is within that range by designing a reasonable filter. Op07 arbitrary-precision arithmetic amplifier has low input polarization, low temperature drift, low noise and long-term stability. In this article, Op07 is selected to design a second-order active bandpass filter. The filtered signal still has some interference. The amplified signal after digital filtering cannot be transmitted to the scanning interface STM 32 in the negative direction. In this article, LM358 is used to design a boost circuit to control the zero degree and polarity of the signal received by the sensor.

### 2.3 Data modeling and prediction

The intelligent MAC system in this article can summarize the collected information, not only analyzing the parameter settings during the analysis process, but also analyzing the trend, correlation, and statistical characteristics of key indicators.

During the fermentation process, sampling information is often affected by various interferences, model degradation, sensor failures, and errors in the data collection system. Impurities in normal information typically manifest as high-frequency noise, anomalies, drift, distortion, and leakage. Therefore, it is necessary to preprocess the data to ensure that effective information falls into the most sensitive input area of the model as much as possible.

The main task of preprocessing is to extend the 3D (three-dimensional) dataset $X_{i}\left(IJK_{i}\right)$ . I represents the number of batches in the container; J represents the number of process variables; K_i_ represents the length of sampling point i.

This article combines the previous separation process steps to extend 3D data to 2D (two-dimensional) data fields. The fermentation ( $x_{i}\left(K_{i}J\right)$ expression) process of each batch of yeast is extended to the following form, and the superscript numbers represent different production stages:

$$X_{i}\left(K_{i}J\right)=\left[x_{i}^{1T}\left(K_{i}^{1}J\right)x_{i}^{2T}\left(K_{i}^{2}J\right)x_{i}^{3T}\left(K_{i}^{3}J\right)x_{i}^{4T}\left(K_{i}^{4}J\right)\right]$$ (1)

This method is used to extend all training data to two-dimensional modeling of matrix data expressions:

$$X\left(\sum_{i=1}^{I}K_{i}J\right)={\left[X_{1}\left(K_{1}J\right)X_{2}\left(K_{2}J\right)\cdots X_{I}\left(K_{I}J\right)\right]}^{T}$$ (2)

When using data-based models, noise detection often leads to significant errors in model evaluation. This article eliminates detection noise by moving windows on blocks, linear filtering, and data scaling. By default, the window block movement is N data points. The model has been trained regularly, expressed as:

$$F_{new}=L\left(f_{init},D_{mw}\right)$$ (3)

In the formula: D_mw_ is the latest N sampled data points; F_new_ is the new model; f_init_ is a modeling method; L is the training data method. An easy to implement linear FIR (finite impulse response) is used to smooth the signal by adjusting the weighted sum in the window, that is, the filter coefficients.

$$x_{t,k}^{d}=\frac{1}{N}\sum_{i=1}^{N}\alpha_{i}x_{t-i,k}$$ (4)

In the formula: α_i_ is the filter coefficient; x_t-i,k_ is the variable data to be smoothed.

In this paper, the relevance vector machine algorithm is used to determine the predictive quality model of each stage of the fermentation process. The relevance vector machine algorithm is a nonlinear modeling method, which has good generalization ability and provides better prediction than the traditional nonlinear modeling method with limited learning data (Liu et al., 2020; Fattahi, 2021). Initially, the algorithm assumes that each stage contains a set of learned data pairs consisting of input process variables and quality variables, denoted as follows.

$$\left\{x^{s},y^{s}\right\}=\left\{x_{i}^{s},y_{i}^{s}\right\}$$ (5)

The non-linear variable relationship is represented as:

$$y^{s}=f\left(x^{s},w^{s}\right)+\epsilon$$ (6)

In the formula, w^s^ is a weighted coefficient vector that follows a Gaussian distribution and satisfies the following formula:

$$p\left(w^{s}\left|\alpha^{s}\right.\right)={\left(2\pi \right)}^{-\left(m+1\right)/2\Pi_{j=0}^{m}{\left(\alpha_{j}^{s}\right)}^{1/2}\times \exp\left(-\frac{1}{2}\alpha_{j}^{s}{\left(w_{j}^{s}\right)}^{2}\right)}$$ (7)

In the formula: α^s^ is a hyperparameter. By optimizing the following formula, the optimal value of α^s^ can be obtained, which is:

$$\text{maxL}\left(\alpha^{s},\delta_{\varepsilon }^{2}\right)=\text{maxlg}\left\{p\left(y^{s}\left|x^{s},\alpha^{s},\delta_{s,\varepsilon }^{2}\right.\right)\right\}$$ (8)

Then the posterior probability of the weighted vector w^s^ is calculated as follows with Formula (9):

$$p\left(w^{s}\left|y^{s},\alpha^{s},\delta_{s,\varepsilon }^{2}\right.\right)={2\pi }^{-\left(m+1\right)/2}{\left|\sum s\right|}^{-1/2}\times \exp\left[-\frac{1}{2}{\left(w^{s}-\mu^{s}\right)}^{T}\sum_{s}^{-1}\left(w^{s}-\mu^{s}\right)\right]$$ (9)

The variance and mean values in the formula can be expressed as:

$$\sum s={\left[\delta_{s,\varepsilon }^{-2}\tau^{T}\left(x^{s}\right)\tau \left(x^{s}\right)+A^{s}\right]}^{-1}$$ (10)

$$\mu^{s}=\delta_{s,\varepsilon }^{-2}\sum s\tau^{T}\left(x^{s}\right)y^{s}$$ (11)

In the formula: $\tau \left(x^{s}\right)$ is the kernel function. The final value of phase mass prediction is represented as the weighted sum of kernel functions, namely:

$$y^{s}=f\left(x^{s},w^{s}\right)+\epsilon =\sum_{i=1}^{R_{V}}{\left(w_{i}^{s}\right)}^{T}K\left(x^{s},x_{j}^{s}\right)+w_{0}^{s}+\epsilon =w^{s}\varphi \left(x^{s}\right)+\epsilon$$ (12)

In the formula, R_V_ is the number of correlation vectors.

This article establishes a mathematical model to describe the relationship between the sensor signals of water-soluble conjugated polymers and key indicators of the fermentation process. The established model is used for prediction and control, and fermentation conditions are adjusted based on real-time collected signals to achieve intelligent control of the fermentation process.

## 3 Evaluation of intelligent MAC effect

In order to improve the MAC effect of food microbial fermentation process, this article applied water-soluble conjugated polymers to sensors and conducts intelligent MAC of food microbial fermentation process. The effect of intelligent MAC of food microbial fermentation process was analyzed by means of laboratory experiments, data analysis tests and practical application validation. The laboratory experiments analyzed the differences between the intelligent MAC system and the traditional method in terms of product yield and fermentation rate under controlled conditions. The data analysis experiment is based on the collected experimental data, using statistical methods and relevant models to analyze the effectiveness and advantages of intelligent MAC systems. Practical application verification: in a real production environment, an intelligent MAC system was applied and compared with traditional methods to evaluate its performance and effectiveness in practical applications. These three steps were followed in order to determine the effectiveness of water-soluble conjugated polymers for practical applications.

### 3.1 Laboratory experimental evaluation

According to the characteristics of fermentation process, it can be divided into three categories: batch fermentation, continuous fermentation and fed-batch culture. Intermittent fermentation refers to a fermentation method that does not exchange materials with the outside world, except for adding acid-base solutions for aeration and acid-base adjustment during the fermentation process. Its disadvantages are long production cycles, high consumption, and low output. Continuous fermentation refers to a fermentation method in which fresh fermentation broth is continuously added to the reactor and continuously discharged to maintain vigorous and stable microbial growth and metabolic activity. Compared with intermittent fermentation, the probability of foreign bacterial contamination is higher and the nutrient utilization rate is lower. Fed-batch culture is a transitional type between batch fermentation and continuous fermentation. This fermentation is a fermentation method similar to batch fermentation. During the process of batch fermentation, nutrient limited medium is added regularly or continuously, and the fermentation is stopped until the required fermentation products reach a certain amount. This method is suitable for various fermentation processes, such as yeast, amino acids, antibiotics, enzymes, organic acids, etc. It combines the advantages of batch fermentation and continuous fermentation, and overcomes the drawbacks of both. The fermentation method selected in this paper is fed-batch culture.

**FIGURE 2:**
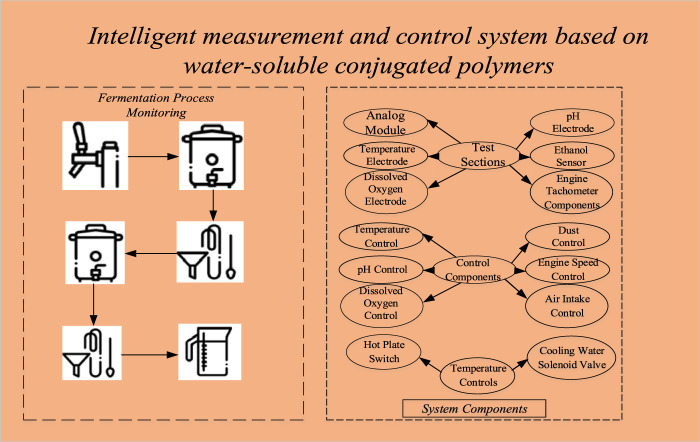
Composition of fermentation intelligent MAC system.

In this paper, a computer is used as a fermentation system monitor to carry out on-line monitoring of the fermentation process through fieldbus and programmable logic controller, such as real-time display of fermentation parameters, data management, data analysis, system parameter setting, and recipe loading. The detection instrument, actuator, and fermentation tank form a multi-loop control system that can determine parameters such as temperature, pH, dissolved oxygen, rate, and ethanol concentration online, and monitor these parameters in real-time. The specific composition of the fermentation intelligent MAC system can be seen in Figure 2:

The test part includes simulation module, temperature electrode, dissolved oxygen electrode, pH electrode, ethanol sensor and engine tachometer element. The control components include temperature control, pH control, dissolved oxygen control, dust control, engine speed control, and intake control. The temperature control device consists of a hot plate switch and a cooling water solenoid valve. When the temperature of the fermentation tank exceeds the set value, the system automatically opens the electromagnetic valve to the cooling water to achieve the purpose of cooling the fermentation tank. When the temperature drops below the specified point, the system turns on the heating disc switch and closes the solenoid valve. To avoid overheating or excessive heat due to system malfunction, the cooling water solenoid valve and heating plate on or off can be set manually.

The pH control consists of an acid pump and an alkaline pump controlled by a 24V/3W micro motor. If the pH is higher than a given value, the system could automatically activate the auxiliary acid pump, which delivers the acid to the fermenter in a time-proportional manner, thus adjusting the pH. In addition, there are both automatic and manual methods to control the addition of acids and bases.

Dissolved oxygen control includes manually adjusting the compressor intake and engine speed. A flow meter can be used to measure the air flow rate, and a compressor sends air into the fermentation tank under a certain pressure. In addition, the dissolved oxygen in the reservoir supply medium can be adjusted by changing the engine speed.

The motor speed is controlled by a frequency converter, which consists of a gearbox, controller, frequency converter, and motor.

The defoaming control consists of an anti-foam electrode and a sliding pump with anti-foam. Control modes include manual and automatic modes. If the defoaming electrode automatically detects excessive foam, the system could start the peristaltic pump and add defoamer in proportion to time.

The supply control device consists of a sliding supply pump. The control method can be executed in proportion to time, or other control strategies can be added as needed.

**FIGURE 3:**
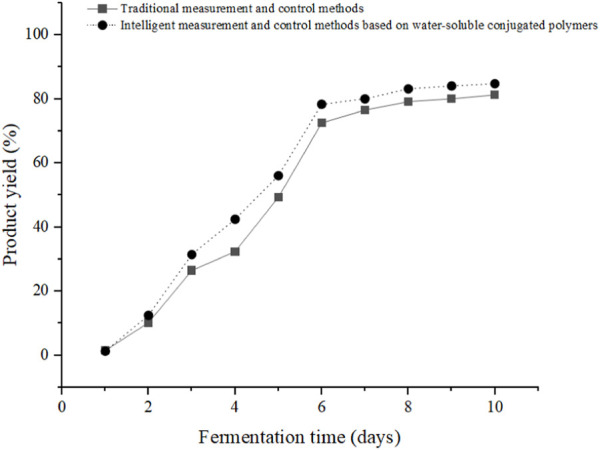
Trend of product yield over time under different MAC methods.

This article analyzes the fermentation process using yeast brewing technology. In an environment with oxygen, yeast converts glucose into water and carbon dioxide. Under anaerobic conditions, glucose is decomposed into carbon dioxide and alcohol. During the brewing process, ethanol is retained. The traditional MAC method and the intelligent MAC method based on water-soluble conjugated polymers were compared and analyzed to analyze the change process of product yield with fermentation time during food microbial fermentation under different MAC methods, and the results can be seen in Figure 3:

Figure 3 shows the trend of product yield over time under different MAC methods, where the x-axis represents fermentation time and the y-axis represents product yield (%). The legends from top to bottom are traditional MAC methods and the intelligent MAC method based on water-soluble conjugated polymers used in this article.

During the fermentation process, the product yield showed an overall upward trend over time, showing a rapid upward trend from day 1 to day 6. Although it also showed an upward trend from day 6 to day 10, the upward trend became slower, which was much slower than the previous 6 days. However, compared to traditional MAC methods and the use of intelligent MAC methods based on water-soluble conjugated polymers in this article, it was found that except for the first day when the product yield of the intelligent MAC method based on water-soluble conjugated polymers was lower than that of traditional MAC methods, the product yield of the intelligent MAC method based on water-soluble conjugated polymers was higher than that of traditional MAC methods for the remaining 9 days.

According to Figure 3, it can be seen that the wine fermentation process was basically completed on the 6th day, and the product yield could not change significantly with time. However, the product yield under the intelligent MAC method based on water-soluble conjugated polymers was higher than that under traditional MAC methods, because the intelligent MAC method based on water-soluble conjugated polymers can improve and regulate the fermentation process in a timely manner. According to ethanol content and other indicators, people were advised to increase necessary fermentation conditions, thereby improving product yield.

In the process of yeast brewing, this article compared and analyzed traditional MAC methods with intelligent MAC methods based on water-soluble conjugated polymers. 20 samples were selected, and a group of 10 samples were labeled as 1–10. The experimental group used an intelligent MAC method based on water-soluble conjugated polymers, while the control group used traditional MAC methods to analyze the actual time required for each sample to complete the brewing process under different MAC methods. The results are shown in Table 1:

**TABLE 1:** Fermentation rate under different MAC methods.

Number	Fermentation time of control group (days)	Fermentation time of experimental group (days)
1	6.5	6.2
2	7.6	5.8
3	8.4	6.7
4	7.4	5.4
5	8.5	8.1
6	9.5	5.1
7	9.7	4.6
8	7.5	5.6
9	8.4	6.4
10	9.5	7.5

When using traditional MAC methods for wine fermentation, it was found that it took at least 6.5 days and at most 9.7 days to obtain the final product, with an average consumption of 8.3 days. However, when utilizing a water-soluble conjugated polymer-based intelligent MAC method for wine fermentation MAC, a minimum of 4.6 days and up to 8.1 days were required, with an average consumption of 6.14 days.

Based on the above results, it can be concluded that using intelligent MAC methods based on water-soluble conjugated polymers for wine fermentation can greatly improve the efficiency of wine fermentation and shorten the time required for wine fermentation. On this basis, the intelligent MAC method used for water-soluble conjugated polymers can improve the yield of wine and can be applied to large-scale production of wine.

In the process of food microbial fermentation, energy consumption affected the cost of producing products. Taking wine fermentation as an example, energy efficiency can be used as an important indicator to evaluate energy consumption in wine fermentation. This provided a reference for the widespread application of intelligent MAC methods based on water-soluble conjugated polymers. After improving the MAC methods, the energy consumption during the fermentation process has also attracted people’s attention. Based on this, this article analyzed the energy utilization efficiency, and the results are shown in Table 2:

**TABLE 2:** Energy utilization efficiency of fermented products under two MAC methods.

Number	Fermentation time of control group (%)	Fermentation time of experimental group (%)
1	63.5	89.5
2	65.5	85.5
3	68.4	87.6
4	69.4	83.5
5	64.8	86.4
6	68.9	87.4
7	67.1	83.5
8	63.4	82.4
9	65.9	89.4
10	62.4	90.5

The energy utilization efficiency of the control group samples did not exceed 70%, with sample 10 having the lowest energy utilization efficiency of 62.4%. Sample 4 had the highest energy utilization rate of 69.4%. The energy utilization efficiency of the experimental group samples exceeded 80%, with sample 8 having the lowest energy utilization efficiency of 82.4%. Sample 10 had the highest energy utilization rate of 90.5%.

There is a significant difference in the energy utilization rate between the two types of MAC samples, but it cannot be denied that the overall use of intelligent MAC methods based on water-soluble conjugated polymers for fermentation product MAC can improve the energy utilization rate of the product. Higher energy utilization indicates a significant reduction in heat loss and more energy for wine fermentation.

### 3.2 Data evaluation test

**FIGURE 4:**
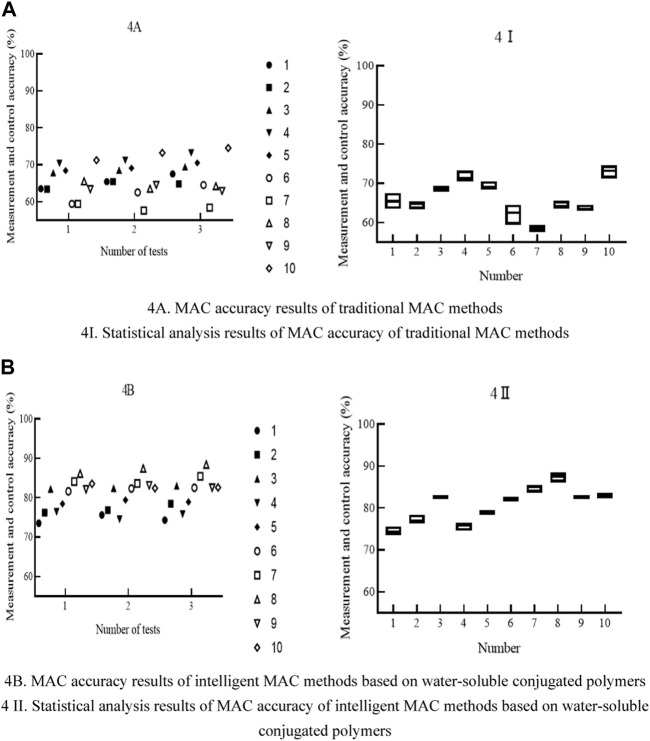
MAC accuracy results and statistical analysis results of two MAC methods. **(A)** Accuracy of traditional MAC methods. **(B)** Accuracy of intelligent MAC methods based on water-soluble conjugated polymers.

The MAC accuracy plays an extremely important role in the intelligent fermentation process of food microorganisms. Only with higher MAC accuracy can the fermentation process be accurately measured and controlled, thereby improving the fermentation rate and completing the fermentation process faster. There is a certain gap in the MAC accuracy of the two MAC methods. In order to analyze the difference in MAC accuracy between the two, the ethanol content of 20 samples was measured three times in this paper and the results were recorded to Figure 4.

In Figures 4, 4A shows the MAC accuracy results of traditional MAC methods; Figure 4I shows the statistical analysis results of MAC accuracy of traditional MAC methods; Figure 4B shows the MAC accuracy results of intelligent MAC methods based on water-soluble conjugated polymers; Figure 4II shows the statistical analysis results of MAC accuracy of intelligent MAC methods based on water-soluble conjugated polymers. The abscissa of Figures 4A,B represents the number of tests, and the ordinate represents the MAC accuracy. The legend shows each test sample from top to bottom. The abscissa of Figures 4I,II represents each sample, and the ordinate represents the MAC accuracy.

The MAC accuracy of traditional MAC methods was within the range of 55%–80%, while the MAC accuracy of intelligent MAC methods based on water-soluble conjugated polymers was within the range of 70%–90%. Comparing the two MAC methods, it was found that the MAC accuracy of traditional MAC methods was greater than that of intelligent MAC methods based on water-soluble conjugated polymers.

Comparing the MAC accuracy of two MAC methods, it was found that the intelligent MAC method based on water-soluble conjugated polymers had high MAC accuracy. The reason for this was that the intelligent MAC method of water-soluble conjugated polymers used high-precision sensors, and the application of water-soluble conjugated polymers also enhanced the sensitivity of sensors. Therefore, the application of intelligent MAC methods based on water-soluble conjugated polymers can improve MAC accuracy.

In order to analyze the performance characteristics of the intelligent MAC method based on water-soluble conjugated polymers designed in this paper, the intelligent MAC system based on water-soluble conjugated polymers and the traditional MAC system were comparatively analyzed from the perspectives of the system’s timeliness of testing, stability of testing, rapidity of testing, and security of control. The system’s performance was tested at the time of the two systems in measuring and controlling different fermentation samples, and the results can be seen in Table 3:

**TABLE 3:** Performance advantages of MAC systems.

MAC systems	Number	Test timeliness(%)	Test stability(%)	Test Rapidity(%)	Control safety(%)
Intelligent MAC system based on water-soluble conjugated polymer	1	76.5	76.8	72.8	75.9
	2	75.8	78.4	76.4	78.4
	3	78.1	79.4	78.1	76.8
	4	76.8	76.4	79.2	72.8
	5	75.1	78.1	75.8	79.5
	6	73.4	79.6	76.8	78.4
	7	76.1	78.7	79.1	77.6
	8	78.5	75.4	75.8	78.5
	9	76.7	79.4	76.8	83.5
	10	75.4	76.5	74.5	80.1
Conventional MAC system	1	60.5	56.4	63.1	62.4
	2	63.4	57.4	64.7	57.5
	3	58.4	58.1	63.4	58.4
	4	57.2	56.4	68.7	62.1
	5	56.1	58.8	65.1	60.7
	6	53.7	56.8	68.2	59.4
	7	58.1	52.4	67.1	58.1
	8	57.8	62.4	61.5	57.5
	9	54.8	63.4	69.4	61.3
	10	58.7	57.3	62.5	62.5

**FIGURE 5:**
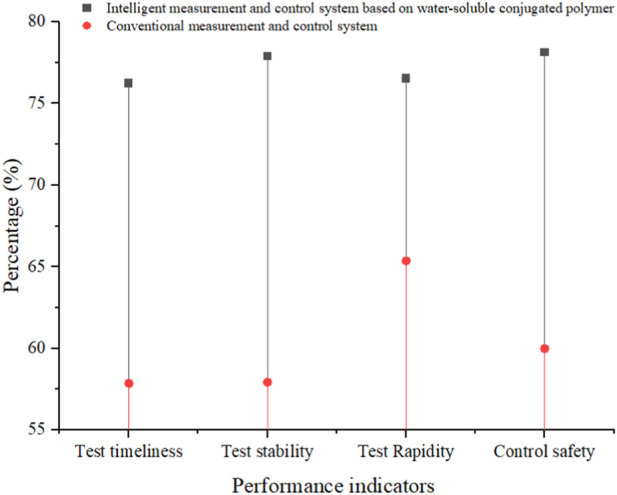
Performance of two MAC systems.

Based on the above results, the average performance indicators of two MAC systems were calculated, and the results were retained to two decimal places. The results can be seen in Figure 5:

The x-axis in Figure 5 represents the system performance indicators selected in this article, while the y-axis represents the percentage of the final result. The illustrations from top to bottom are intelligent MAC systems based on water-soluble conjugated polymers and traditional MAC systems.

The average test timeliness, stability, speed, and control safety of traditional MAC systems were lower than those of intelligent MAC systems based on water-soluble conjugated polymers. Among them, the average test timeliness of traditional MAC systems was 18.37% lower than that of intelligent MAC systems based on water-soluble conjugated polymers; the average test stability of traditional MAC systems was 19.93% lower than that of intelligent MAC systems based on water-soluble conjugated polymers; the average testing speed of traditional MAC systems was 11.16% lower than that of intelligent MAC systems based on water-soluble conjugated polymers; the average control safety of traditional MAC systems was 18.16% lower than that of intelligent MAC systems based on water-soluble conjugated polymers.

The performance of traditional MAC systems is significantly inferior to that of intelligent MAC systems based on water-soluble conjugated polymers. The reason for this is that the application of water-soluble conjugated polymers has made MAC during the fermentation process more agile and fast, and the application of sensors based on water-soluble conjugated polymers has also improved the accuracy of testing. People no longer need to repeatedly measure microbial parameters during the fermentation process, which greatly saves MAC time, improves MAC efficiency, and thus accelerates the entire fermentation process.

### 3.3 Practical application verification

**FIGURE 6:**
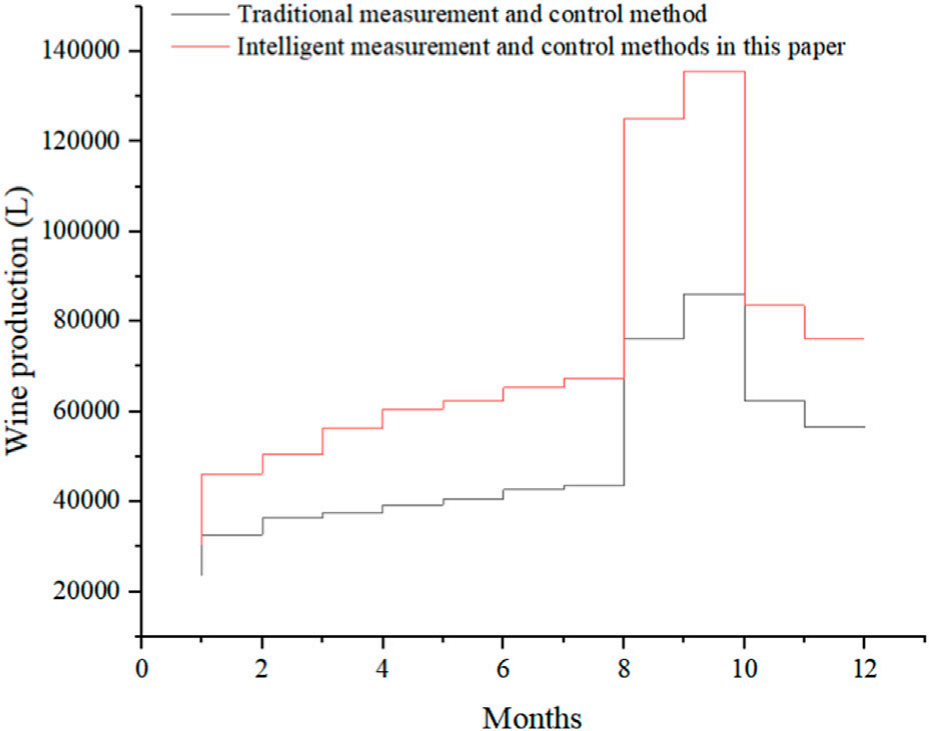
Winery’s wine production from January to December 2022.

In order to verify the application effect of the intelligent MAC method based on water-soluble conjugated polymers designed in this article in actual production, this article took a winery L as an example to analyze the fermentation process of batch production wine. Under different MAC methods, the winery’s brewing process adopted two different MAC methods for MAC, and the raw materials and other conditions before fermentation were basically the same under the two MAC methods. Under these conditions, the wine production under two MAC methods from January to December 2022 was recorded, as shown in Figure 6:

The x-axis in Figure 6 represents different months, and the y-axis represents wine production. The legends from top to bottom are the traditional MAC methods and the intelligent MAC methods in this article. During the entire year of wine production, the wine production using traditional MAC methods was lower in each month than that using intelligent MAC methods based on water-soluble conjugated polymers. Wine production under the two measurement methods showed a slow upward trend from January to August. In September, wine production increased dramatically, with more production in October than in September, followed by a downward trend in November and December.

Based on the above analysis, it can be seen that using intelligent MAC methods based on water-soluble conjugated polymers can greatly improve the production of fermentation products. Moreover, in the actual application process of wineries, it has been found that using intelligent MAC methods based on water-soluble conjugated polymers can improve the production of wine compared to traditional MAC methods. The application of the intelligent MAC method based on water-soluble conjugated polymers in the process of food microbial fermentation in this article is practical and effective. The wine production in September using both MAC methods is nearly twice that of August, and the reason why October is more than September is that these 2 months are the main months for grape ripening. Due to the abundant raw materials for brewing, the wine production also increases rapidly, which indirectly indicates that wine brewing has a certain seasonality.

In addition to investigating and analyzing winery L, this paper also analyzed 10 high-profile wineries, which were labeled A-J, and took the same approach as above to analyze the wines from the wineries. The main purpose of this survey was to evaluate the quality of wine produced using two different MAC methods. The evaluation method was expert tasting, and a group of 10 experts was formed to evaluate the wine of each winery from three aspects: aroma, color, and taste. The full score for aroma was 25 points; the full score for color was 25 points; the full score for taste was 50 points. The scoring results of each expert were added together to obtain the average value, and the comprehensive wine rating was finally summarized. The results are shown in Table 4:

**TABLE 4:** Quality evaluation results of wine.

MAC systems	Wineries	Aroma	Colour	Flavour	Total score
Intelligent MAC system based on water-soluble conjugated polymer	A	23.5	23.9	47.5	94.9
	B	24.5	24.4	46.4	95.3
	C	24.1	23.7	45.2	93
	D	23.7	23.6	46.5	93.8
	E	23.6	24.6	48.5	96.7
	F	22.9	23.1	44.5	90.5
	G	23.6	24.6	42.7	90.9
	H	24.5	24.5	41.9	90.9
	I	24.1	23.8	42.6	90.5
	J	23.8	23.5	43.5	90.8
Conventional MAC system	A	20.1	20.5	40.6	81.2
	B	19.8	18.5	39.5	77.8
	C	16.8	19.3	38.7	74.8
	D	18.5	17.5	36.8	72.8
	E	17.9	18.4	37.8	74.1
	F	19.6	18.3	34.8	72.7
	G	20.4	18.8	36.1	75.3
	H	21.6	16.4	38.7	76.7
	I	22.4	18.3	39.5	80.2
	J	21.5	19.4	41.5	82.4

The use of intelligent MAC methods based on water-soluble conjugated polymers can not only improve the aroma, color, and taste of wine compared to traditional MAC methods, produce more popular wine, but also improve the quality of wine from different wineries. This indicates that intelligent MAC methods based on water-soluble conjugated polymers not only have a positive promoting effect on the wine brewing of a certain winery, and it is applicable to all wineries.

When using traditional MAC methods, Winery F had the lowest score of 72.7 points, while Wine J had the highest score of 82.4 points. When using an intelligent MAC method based on water-soluble conjugated polymers, the comprehensive score of each winery’s wine exceeded 90 points. The comprehensive scores of Winery I and Winery F were consistent, both of which were 90.5 points. However, they were at a lower score among the 10 wineries surveyed, with Winery E having the highest comprehensive score of 96.7 points. The reason for the analysis may be that these 10 wineries are located in different locations and use different grape ingredients, resulting in significant differences in the quality of the final wines they produce.

Among the 10 selected wineries, Winery A’s comprehensive score increased by 13.7 points; Winery B’s comprehensive score increased by 17.5 points; Winery C’s comprehensive score increased by 18.2 points; Winery D’s comprehensive score increased by 21 points; Winery E’s comprehensive score increased by 22.6 points; Winery F’s comprehensive score increased by 17.8 points; Winery G’s comprehensive score increased by 15.6 points; the comprehensive score of Winery H increased by 14.2 points; Winery I increased by 10.3 points; Winery J increased by 8.4 points. Based on this result, it can be concluded that Winery E has the largest increase in overall score, while Winery J has the smallest increase in overall score. The reason for this phenomenon may be that different judges have different preferences for wine, so their ratings for different wines vary greatly.

## 4 Discussion

During the laboratory experimental analysis, it was found that although the product yield of the intelligent MAC method based on water-soluble conjugated polymers was lower than that of traditional MAC methods in the initial fermentation process, the product yield of the intelligent MAC method based on water-soluble conjugated polymers quickly surpassed that of traditional MAC methods in the subsequent fermentation process. The use of intelligent MAC methods based on water-soluble conjugated polymers for wine fermentation can greatly improve the efficiency of wine fermentation and the utilization of energy during the fermentation process. This indicates that the intelligent MAC method based on water-soluble conjugated polymers is not only conducive to improving product yield, fermentation efficiency, and fermentation product yield, but also can maximize energy utilization and improve energy utilization. This is beneficial for promoting the entire fermentation process, indicating the feasibility of utilizing intelligent MAC methods based on water-soluble conjugated polymers in the laboratory analysis stage.

The data analysis experiment mainly found that the MAC accuracy of the intelligent MAC method based on water-soluble conjugated polymers is much higher than that of traditional MAC methods, and its testing timeliness, stability, speed, and control safety are higher than those of traditional MAC methods. The data analysis results of the intelligent MAC method based on water-soluble conjugated polymers show that this method has excellent advantages and can be applied to the fermentation process. This further verifies the effectiveness of the intelligent MAC method based on water-soluble conjugated polymers in the application of fermentation processes.

In the analysis of practical application verification, it was found that wineries using intelligent MAC methods based on water-soluble conjugated polymers can produce more wine than conventional MAC methods. Moreover, using this method to analyze the quality of different wineries, it was found that the wine quality of wineries under this method is better than that of wineries under traditional MAC methods. The use of intelligent MAC methods based on water-soluble conjugated polymers in practical applications in wineries has effectively improved wine production and quality, indicating that the next step can be to expand the scope of application in the fermentation industry.

Based on the comprehensive analysis of laboratory experiments, data analysis experiments, and practical application verification, using intelligent MAC methods based on water-soluble conjugated polymers has extremely important advantages compared to traditional MAC methods. Its application prospects in food microbial fermentation processes are broad, and it can be expanded to apply to more food enterprises, which also provides a reference path for the development of food enterprises.

## 5 Conclusion

In order to improve the accuracy of MAC during food microbial fermentation, this paper designed a sensor using water-soluble conjugated polymers and designed an intelligent MAC system based on the water-soluble conjugated polymer-based sensor. This intelligent MAC method was applied to food microbial fermentation and brewing, and compared with traditional MAC methods. A comparative analysis of the two MAC methods was conducted from the perspectives of laboratory experiments, data analysis experiments, and practical application validation. After verification, it was found that the MAC method based on water-soluble conjugated polymers not only has better system advantages, but also greatly improves the yield of wine in practical applications. The MAC method based on water-soluble conjugated polymers is effective and feasible, and can be applied to food factories engaged in food microbial fermentation on a larger scale, thereby benefiting more people.

## Data Availability

The original contributions presented in the study are included in the article/Supplementary Material, further inquiries can be directed to the corresponding authors.
